# Accessing the Anisotropic Nonthermal Phonon Populations in Black Phosphorus

**DOI:** 10.1021/acs.nanolett.1c01786

**Published:** 2021-07-19

**Authors:** Hélène Seiler, Daniela Zahn, Marios Zacharias, Patrick-Nigel Hildebrandt, Thomas Vasileiadis, Yoav William Windsor, Yingpeng Qi, Christian Carbogno, Claudia Draxl, Ralph Ernstorfer, Fabio Caruso

**Affiliations:** † Fritz Haber Institute of the Max Planck Society, Faradayweg 4-6, 14195 Berlin, Germany; ‡ Department of Mechanical and Materials Science Engineering, Cyprus University of Technology, P.O. Box 50329, 3603 Limassol, Cyprus; ∥ Institut für Physik and IRIS Adlershof, Humboldt-Universität zu Berlin, Berlin, Germany; ⊥ Institut für Theoretische Physik und Astrophysik, Christian-Albrechts-Universität zu Kiel, D-24098 Kiel, Germany

**Keywords:** ultrafast electron diffraction, first-principles calculations, layered materials, black phosphorus, electron−phonon coupling

## Abstract

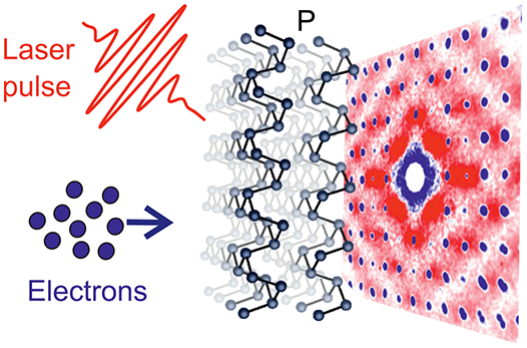

We combine ultrafast electron diffuse scattering experiments and first-principles calculations of the coupled electron–phonon dynamics to provide a detailed momentum-resolved picture of lattice thermalization in black phosphorus. The measurements reveal the emergence of highly anisotropic nonthermal phonon populations persisting for several picoseconds after exciting the electrons with a light pulse. Ultrafast dynamics simulations based on the time-dependent Boltzmann formalism are supplemented by calculations of the structure factor, defining an approach to reproduce the experimental signatures of nonequilibrium structural dynamics. The combination of experiments and theory enables us to identify highly anisotropic electron–phonon scattering processes as the primary driving force of the nonequilibrium lattice dynamics in black phosphorus. Our approach paves the way toward unravelling and controlling microscopic energy flows in two-dimensional materials and van der Waals heterostructures, and may be extended to other nonequilibrium phenomena involving coupled electron–phonon dynamics such as superconductivity, phase transitions, or polaron physics.

Black phosphorus (BP) exhibits a tunable band gap in the mid-IR,^1−3^ high carrier mobilities,^4−6^ and a layered crystal structure. These features make it a versatile platform to explore novel device concepts, such as field-effect transistors, saturable absorbers, and polarization-sensitive photodetectors.^2,4,5,7−9^ The pronounced crystal structure anisotropy of BP further gives rise to highly anisotropic macroscopic properties, as exemplified by its thermal^10−12^ and electrical conductivities,^1,5,13,14^ as well as its optical response.^5,15−17^

Since practical applications based on these properties invariably exploit nonequilibrium states of either the lattice or hot carriers, it is desirable to attain a detailed understanding of the ultrafast dynamics of electronic and vibrational degrees of freedom in BP. Following photoexcitation, hot carriers relax to the band edges by transferring their excess energy to the lattice via the emission of phonons, which triggers coupled carrier-lattice dynamics. Optical and photoemission spectroscopies have been employed extensively to investigate carrier-phonon scattering channels and their influence on the carrier dynamics in BP.^14,18−25^ While these techniques provide direct information on the electrons, the lattice dynamics can only be inferred indirectly through its effects on the electronic structure. Ultrafast electron diffuse scattering (UEDS), conversely, circumvents these limitations and complements optical and photoemission spectroscopies. UEDS provides direct access to lattice dynamics and electron–phonon scattering processes with time and momentum resolution.^26−32^ Because of its sensitivity to both electron–phonon and phonon–phonon scattering processes in reciprocal space, UEDS is thus well-suited to establish a microscopic picture of the energy flows between hot electrons and the BP lattice.

Here, we combine UEDS experiments with ab initio calculations to provide a momentum-resolved picture of nonradiative energy flows in photoexcited BP. We observe that strongly anisotropic nonthermal phonon populations are established throughout the first picoseconds of the dynamics, and thermal equilibrium is only re-established by anharmonic decay pathways (phonon–phonon coupling) on time scales of the order of 50–100 ps. To unravel the origin of the nonequilibrium lattice dynamics and its signatures in UEDS experiments, we conduct first-principles calculations of the coupled electron–phonon dynamics based on the time-dependent Boltzmann formalism, whereby electron–phonon and phonon–phonon scattering processes are explicitly taken into consideration. Calculations of the structure factor further enable a direct comparison with the experimental data. Our findings reveal how band-structure anisotropies profoundly influence the decay path of photoexcited carriers and are at the origin of nonthermal phonon populations.

## Results and Discussion

**Figure 1:**
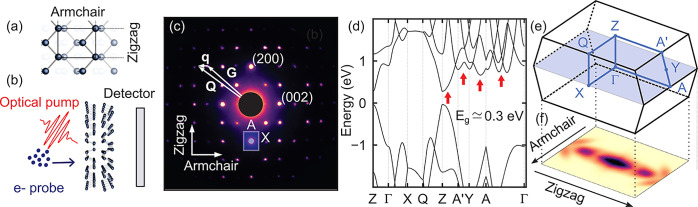
(a) Top view of the BP crystal lattice. (b) Schematic illustration of ultrafast electron diffuse scattering, with side view of the BP crystal lattice. (c) Representative transmission diffraction pattern of BP. The Brillouin zone can be drawn around each Bragg peak, as illustrated by the rectangle over the (2̅00) reflection. An arbitrary position in reciprocal space, **Q**, can always be expressed as **G** + **q**, where **G** is a reciprocal lattice vector and **q** the phonon wavevector. (d) Electronic band structure as obtained from density functional theory (DFT). A scissor rigid shift of 0.2 eV has been applied to the conduction manifold to match the experimental band gap (*E*_g_ ≃ 0.3 eV).^33^ (e) Brillouin zone and high-symmetry points of BP. The blue shading marks the region of reciprocal space probed by our UEDS measurements. (f) Fermi–Dirac occupations of photoexcited carriers for momenta in the Q-Z-A′ plane in the BZ.

The layered orthorhombic crystal structure of BP is illustrated in Figures 1a and 1b from the top and side view, respectively, whereas its Brillouin zone (BZ) and main high-symmetry points (labeled according to the convention of ref (34)) are reported in Figure 1e. The equilibrium electron diffraction pattern of Figure 1c provides a direct view of the reciprocal lattice for momenta within the X-Γ-A plane in the BZ (shaded blue plane in Figure 1e). High-intensity features arise for transferred momenta matching the reciprocal lattice vectors **G**, according to Bragg’s law. These measurements are consistent with previous TEM experiments.^35^ Besides the pronounced anisotropy of the BP crystal lattice, signatures of anisotropy also manifest themselves in the electronic properties.

The electronic band structure, obtained from density functional theory (DFT) and illustrated in Figure 1d, exhibits a direct gap at the Z-point and a conduction band characterized by several local minima in the vicinity of the Y, A, and A′ high-symmetry points. The local minima in the conduction band thus involve crystal momenta with an in-plane component directed primarily along the zigzag direction. Conversely, no local minima arise in the conduction band along Γ-X and Z-Q (armchair direction). The anisotropic character of the band structure is shown below to influence profoundly the nonequilibrium dynamics of electrons and phonons in BP, leading to the emergence of a striking anisotropy in the phonon population following photoexcitation.

### Ultrafast Electron Diffuse Scattering Measurements

To investigate the nonequilibrium lattice dynamics of BP with momentum and time resolutions, we perform UEDS measurements on a free-standing thin film of BP. The sample has an estimated thickness of 39 ± 5 nm and has been obtained by mechanical exfoliation of a bulk crystal. In UEDS, a laser pulse is employed to drive the system into an excited electronic state. After a time delay *t*, the sample is probed by an electron pulse, which diffracts off the lattice. The diffraction pattern generated by this procedure provides a direct probe of the nonequilibrium dynamics of the lattice in reciprocal space.^36^ A schematic illustration of the experiment is reported in Figure 1b. Here, the BP flake is photoexcited with a 50 fs light pulse with energy *hν* = 1.61 eV and polarization aligned along the armchair direction. Additional measurements using a pump energy *hν* = 0.59 eV are reported in the Supporting Information. The duration of the electron pulse is estimated to be ∼200 fs. All measurements are performed at the temperature of 100 K. The initial density of photoexcited electrons and holes induced by the pump pulse is estimated to *n*_*e*_ = 7.3 × 10^13^ cm^–2^ (see the Supporting Information).

**Figure 2:**
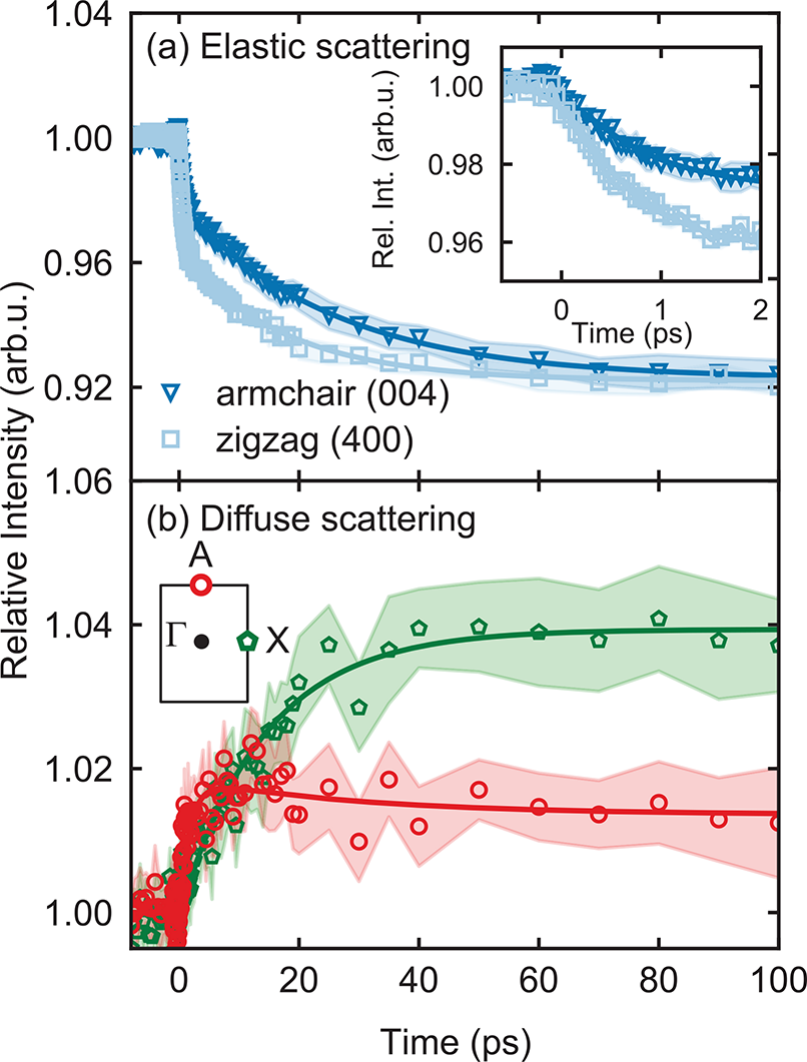
(a) Exemplary anisotropic elastic scattering signals for *zigzag* (squares) and *armchair* reflections (triangles). Inset: zoom into the first 2 ps of the dynamics. (b) Diffuse scattering signal at A (circles) and X (pentagons) around the (400) reflection. The data in both panels are the average over the Friedel pair (e.g., (400) and (4̅00)). The error estimates represent the standard error of the mean signal over multiple delay scans.

Figure 2a illustrates the relative intensity changes of the (400) and (004) Bragg peaks, located along the zigzag (squares) and armchair (triangles) directions, respectively, throughout the nonequilibrium dynamics of the lattice. A clear fingerprint of anisotropic lattice dynamics is revealed by the different time dependence of these elastic scattering signals. The dynamics of both armchair and zigzag reflections are well-captured by biexponential decays, with a fast time constant of 500 fs and a slower time constant of 20 ps. This behavior was described in detail in ref (37), where some of us investigated the dynamics of the Bragg reflections in BP, revealing nonthermal phonon distributions persisting for tens of picoseconds.

To obtain a momentum-resolved picture of the nonequilibrium lattice dynamics of BP, we go beyond the analysis of the elastic scattering signals and we inspect the transient signatures of diffuse (inelastic) scattering processes, as revealed by UEDS. The contribution of the different high-symmetry points to the UEDS intensity can be singled out by dividing the diffraction pattern into BZs around each Bragg reflection peak, as illustrated by the shaded rectangle in Figure 1c for the (2̅00) reflection. Exemplary time-resolved UEDS signals around the (400) Bragg peak are shown in Figure 2b for the A (circles) and X (pentagons) points in the BZ. As diffuse scattering occurs primarily through phonon-induced scattering processes, the signal measured at a given point **q** in the BZ reflects the phonon population with the same momentum.^28,29,31,38−40^ The red curve in Figure 2b indicates the relative intensity of the UEDS signal as a function of time at point A. Similar dynamics are observed at all investigated points A. A biexponential fit to the data yields a rising time constant of 1.7 ± 0.1 ps, followed by a slower relaxation of 30 ± 2 ps. We note that the 1.7 ps time constant does not appear in an elastic scattering analysis. The time evolution of the diffuse signal at X, shown in green in Figure 2b, reveals a drastically different phonon dynamics compared to point A. Fitting with an exponential function yields a time constant of 14.3 ± 0.1 ps. These measurements indicate a striking anisotropy of the transient UEDS intensity in the BZ, suggesting a highly momentum-dependent excitation and relaxation of the lattice following photoexcitation.

**Figure 3:**
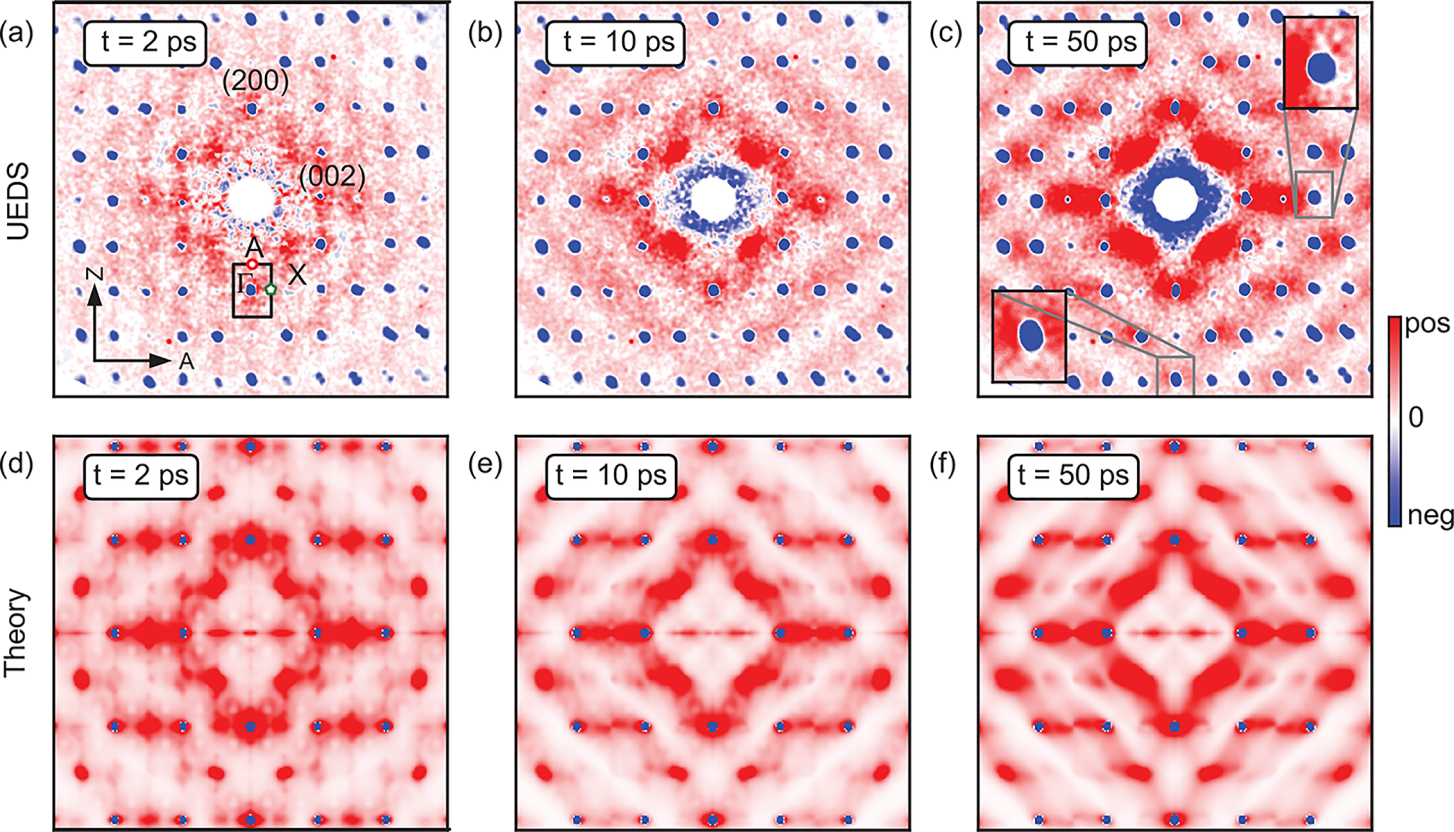
(a–c) Momentum-resolved electron diffraction signals, *I*(**Q**, *t*) – *I*(**Q**, *t* ≤ *t*_0_), at pump–probe delays of 2, 10, and 50 ps. 2-fold symmetrized data,^47^ raw data shown in Supporting Information. The Bragg reflections (blue dots) are negative due to the Debye–Waller effect. The diffuse background (red) qualitatively evolves as a function of pump–probe delay. Selected Brillouin zones are shown in inset for the (004) and the (4̅00) reflections on the 50 ps map. All data are normalized to a common number. (d–f) Simulated nonequilibrium scattering signals at pump–probe delays of 2, 10, and 50 ps. The phonon temperatures are based on the nonthermal model described in the text and shown in Figure 4 (a). All data are normalized to a common number.

A comprehensive view of transient phonon distributions in momentum space is shown in Figures 3a–c, at pump–probe delays of 2, 10, and 50 ps. This set of data demonstrates profound changes in the diffuse scattering signal as pump–probe delay increases, reflecting different phonon populations at different times. While the diffuse pattern at 2 ps is weak and displays faint lines in the Γ–A direction, the diffuse signal at 50 ps is more pronounced, differently shaped, and more anisotropic. A closer inspection of the changes in diffuse scattering signal around Bragg reflections, shown as insets in Figure 3c, reveals high anisotropy between the intensities along the two main crystal axes at 50 ps. These highly anisotropic dynamics within a given BZ, and between BZs, highlight the value of time-resolved diffuse scattering as direct probes of transient nonthermal phonon distributions in momentum space (Figures 3a–c).

### Theoretical Modeling of Nonequilibrium Lattice Dynamics

To gain further insight into the nonequilibrium dynamics of the lattice, we perform first-principles calculations of the coupled electron–phonon dynamics of BP based on the time-dependent Boltzmann equations:^41−56^
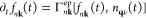
1
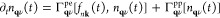
2Here, ∂_*t*_ = ∂/∂*t*, *f*_*n***k**_ denotes the electron distribution function for electron band index *n* and electron momentum **k**, and *n*_**q**ν_ the phonon distribution function for wavevector **q** and branch index ν. Equations 1 and 2 account seamlessly for the effects of electron–phonon and phonon–phonon scattering on the ultrafast dynamics of electrons and phonons with momentum resolution. Radiative recombination and its influence on the electron–phonon dynamics have been neglected in this work. Recent experimental studies estimated radiative processes in BP to occur over time scales of the order of 1 ns.^42−44^ Since these time scales are three (two) orders of magnitude slower than the characteristic time scales of electron–phonon (phonon–phonon) scattering, we expect radiative recombination to be inconsequential for the nonequilibrium lattice dynamics of BP. Electron–electron scattering is further neglected in eqs 1 and 2. Ultrafast experiments for layered materials indicate that, following photoexcitation, electrons thermalize with each others within <60 fs.^45,46^ These findings suggest that electron–electron scattering mostly influences the initial stage of dynamics over time scales, which are shorter than a phonon period. The influence of the electron–phonon interaction on *f*_*n***k**_ (*n*_**q**ν_) is taken into consideration by the collision integral Γ_*n***k**_^ep^ (Γ_**q**ν_^pe^), whereas the phonon–phonon scattering due to lattice anharmonicities is taken into consideration via Γ_**q**ν_^pp^. In short, eqs 1 and 2 have been solved for electron (phonon) momenta within the Q-Z-A′ (X-Γ-A) plane in the BZ by time-stepping the time derivative with intervals of 1 fs for a total simulated time of 100 ps (10^5^ time steps), with the collision integrals being recomputed at each time step. A detailed account of the numerical implementation and explicit expressions for the collision integrals have been reported elsewhere.^41^

As the initial condition for the time propagation, we consider an electronic excited state characterized by a density *n* of electrons and holes excited to the conduction and valence bands, respectively. This state is realized by defining electronic occupations in the conduction band according to *f*_*n***k**_^0^ (μ_*e*_ ,*T*_el_^0^) = [*e*^(ε_*n***k**_ – μ_*e*_/*k*_B_*T*_el_^0^)^ + 1]^−1^. μ_e_ is the chemical potential of the electrons in the conduction band, obtained by solving the integral equation *n* = Ω_BZ_^–1^ ∑_*m*_^cond.^ ∫d**k***f*_*m***k**_^0^ (μ_e_, *T*_el_^0^), where *n* = 7.3 × 10^13^ cm^–2^ is the density of photoexcited carriers estimated in the experiment (see the Supporting Information). A similar treatment is applied to holes in the valence band. The initial electronic temperature *T*_el_^0^ = 7000 K is related to the excess energy of the excited electrons and holes, and is chosen such that the final vibrational temperature of the lattice after thermalization *T*_ph_^fin^ matches the experimental estimate of 300 K. The lattice is initially at thermal equilibrium, with phonon occupations defined according to the Bose–Einstein statistics *n*_**q**ν_^BE^ = [*e*^ℏω_**q**ν_ /*k*_B_*T*_ph_^0^^ – 1]^−1^, at the same temperature of experiments *T*_ph_^0^ = 100 K. Different choices for the shape of the initial electronic excitation are not expected to induce significant changes for the nonequilibrium dynamics of the lattice: the thermalization of excited electronic states in layered materials occurs within few tens of femtoseconds,^45,46^ thus, restoring electronic occupations according to a Fermi–Dirac function before the onset of electron–phonon scattering.

**Figure 4:**
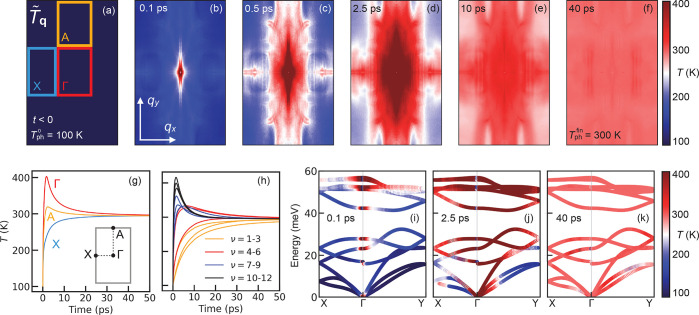
(a) Effective vibrational temperature *T̃*_**q**_ for crystal momenta in the X-Γ-A plane of the Brillouin zone before excitation (*t* < 0), and at several time delays throughout the nonequilibrium dynamics of the lattice (panels (b–f)). (g) Time dependence of *T̃*_**q**_ for momenta around the high-symmetry points Γ (red), A (yellow), and X (blue). Each curve has been obtained by averaging *T̃*_**q**_ for momenta within the regions highlighted in panel (a) at each time step. (h) Time dependence of the branch-resolved vibrational temperature *T*_ν_ (averaged over momentum). ν = 1–3 denote the acoustic branches, ν = 10–12 the highest-energy optical phonons, etc. (i–k) Branch- and momentum-resolved effective vibrational temperatures, superimposed to the phonon dispersion as a color coding, for *t* = 0.1 (i), 2.5 (j), and 40 ps (k).

From the phonon distribution function *n*_**q**ν_(*t*), we calculate the momentum-resolved effective vibrational temperature of the lattice *T̃*_**q**_ = *N*_ph_^–1^ ∑_ν_*T*_**q**ν_, where *N*_ph_ = 12 is the number of phonon branches of BP and *T*_**q**ν_ = ℏω_**q**ν_ {*k*_B_ ln [1 + *n*_**q**ν_(*t*)]}^−1^. In Figure 4a–f, we report *T̃*_**q**_ at different time steps of the coupled electron–phonon dynamics for crystal momenta within the X-Γ-A plane of the BZ (shaded blue in Figure 1(e), corresponding to the plane probed in the UEDS experiments). Before excitation (*t* < 0), the constant temperature *T̃*_**q**_ = 100 K in the BZ reflects thermal equilibrium. At *t* = 0.1 ps, red features in Figure 4(b) indicate the enhancement in the phonon population around Γ (zone center) and along the Γ-A high-symmetry line. This anisotropy becomes more pronounced at later times, as shown in Figures 4c and 4d for *t* = 0.5 and 2.5 ps, respectively. As anticipated above, the origin of this behavior is related to the anisotropy of the valence and conduction bands.

Because of the absence of local minima in the conduction band along the armchair direction (i.e., Γ–X and Z–Q), the photoexcited electrons are constrained to occupy states with crystal momenta along the zigzag direction, i.e., where the available local minima are located (arrows in Figure 1d). This scenario is illustrated by highly anisotropic initial electronic occupations *f*_*n***k**_^0^ in the conduction band, reported in Figure 1f. Because of momentum conservation, carrier relaxation involves phonons with momenta **q** along the Γ–A direction, which are responsible for transitions from the local conduction band minima along Γ–A to the conduction band pocket around the minimum at the Z-point. Conversely, the observed phonon excitations around Γ are responsible for transitions from higher-lying states in the Z-pocket to the conduction band edge at Z and for transitions within the individual pockets at the local minima. Based on this picture, the anisotropic increase of *T̃*_**q**_ in the BZ reflects the phase-space constraints in the electron–phonon interactions, and thus in the relaxation path of photoexcited electrons and holes.

For *t* = 10 ps (Figure 4e), the anisotropy of *T̃*_**q**_ in the BZ is significantly reduced. On these time scales, phonon–phonon scattering, accounted for via Γ^pp^ in eq 2, counteracts the effects of electron–phonon scattering by driving the lattice toward thermal equilibrium. For *t* = 40 ps (Figure 4f), thermal equilibrium is re-established at the temperature *T*_ph_^fin^ = 300 K.

To gain further insight into the anisotropic lattice dynamics, Figure 4g displays the time dependence of *T̃*_**q**_ around the X, Γ, and A regions (obtained by averaging *T̃*_**q**_ over the rectangles in Figure 4(a)) throughout the first 50 ps of the dynamics. For momenta around Γ and A, the temperature reaches a maximum at 1.7 and 2.3 ps, respectively, whereas no maximum is observed around X. These time scales indicate the time required for the electrons to transfer energy to the lattice via electron–phonon scattering. The good agreement with the experimental time constant of 1.7 ps extracted from the rise of the UEDS intensity at *A* (Figure 2b) suggests that transient changes of the UEDS intensities for time scales smaller than 2 ps primarily reflect the energy transfer from the electrons to the lattice driven by electron–phonon coupling.

In Figure 4h, we report the average vibrational temperature for each phonon branch (*T̃*_ν_ = Ω_BZ_^–1^ ∫ d**q***T*_**q**ν_) throughout the first 50 ps, whereas the vibrational temperatures superimposed to the phonon dispersion is illustrated in Figures 4i–k. Because the contribution of each phonon mode to the carrier relaxation is dictated by its coupling strength, modes characterized by stronger coupling provide a preferential decay channel for the excited electrons and, thus, exhibit a higher vibrational temperature throughout the initial stages of the dynamics. In particular, Figures 4h–k indicate that electron relaxation is dominated by high-energy optical phonons, whereby the out-of-phase vibration of P atoms leads to the largest electron–phonon coupling strength.

To inspect directly the influence of the nonequilibrium lattice dynamics on the scattering intensity probed in the UEDS experiments, we conduct first-principles calculations of the structure factor by explicitly accounting for the influence of electron–phonon interactions and anisotropic population of the vibrational modes in the unit cell. Details of the structure factor computations are found in the Supporting Information and elsewhere.^48,49^ The calculated (nonequilibrium) all-phonon structure factor is shown in Figure 3d for *t* = 2 ps. The intensity is relative to equilibrium at 100 K. The calculation agrees well with the experimental UEDS intensity reported in Figure 3a and reproduces the main fingerprints of nonequilibrium lattice dynamics. In particular, the faint vertical high-intensity features connecting the Bragg peaks across different BZ, constituting a clear manifestation of the nonequilibrium state of the lattice, are well-captured by the simulations. The time dependence of the vibrational temperature in the BZ, illustrated in Figure 4, enable us to attribute these features to the higher population of phonons along the Γ–A direction, which, in turn, arises from the primary role played by these phonons in the relaxation of the excited electronic distribution. The calculated UEDS intensities at 10 and 50 ps, shown in Figures 3e and 3f, respectively, capture the emergence of a diamond-shaped diffraction pattern that characterizes the return to thermal equilibrium.

**Figure 5:**
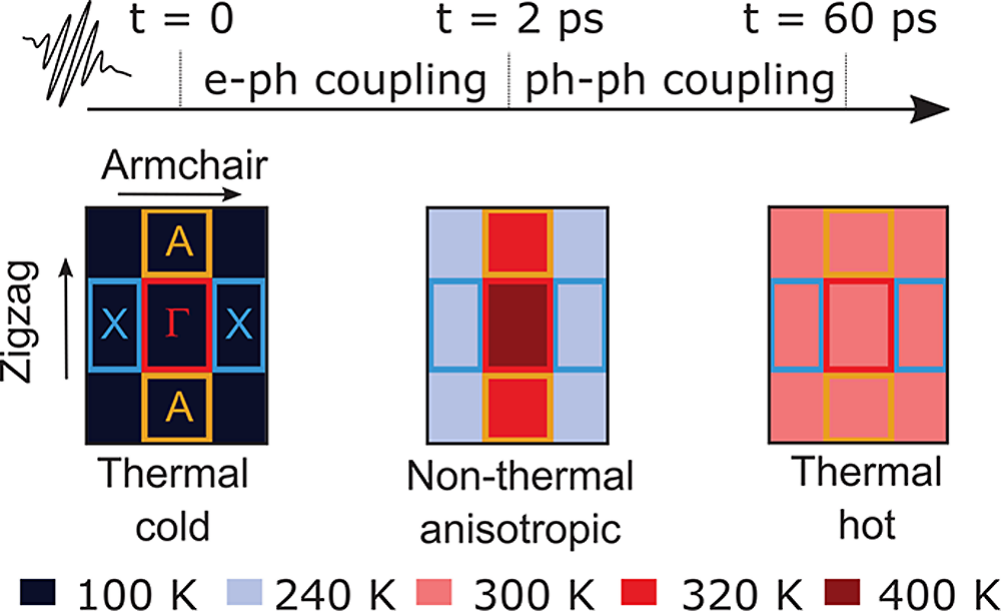
Sketch of the nonequilibrium dynamics and thermalization of the BP lattice following photoexcitation.

These findings enable us to establish the picture sketched in Figure 5 for the lattice thermalization in BP: After the creation of an excited electronic distribution by a laser pulse, electrons (holes) in the conduction (valence) band undergo electron–electron scattering and occupy the band edges according to Fermi–Dirac statistics. This results into a highly anisotropic distribution of photoexcited carriers in the BZ, predominantly populating the Z, Y, A, and A′ pockets. Within 2 ps after photoexcitation, electrons and holes lose their excess energy upon emitting phonons. Momentum selectivity in the phonon emission leads to the primary excitation of phonons with momenta along the zigzag direction of the crystal, driving the lattice into a nonequilibrium regime characterized by a highly anisotropic phonon population in the BZ (Figure 4b–d). Distinctive fingerprints of this regime are visible in the UEDS intensity at *t* = 2 ps (Figure 3a). The ensuing *hot*-phonon population subsequently thermalizes with other lattice vibrations via phonon–phonon scattering, thereby driving the lattice toward thermal equilibrium (i.e., *T*_**q**ν_ = constant) within 50 ps, and leading to the thermalized UEDS intensity reported in Figure 3c. We predict that this thermalization picture remains qualitatively the same for initial temperatures up to at least 500 K (see the Supporting Information).

## Conclusions

We have provided a comprehensive picture of the microscopic energy flows in the crystal lattice of BP following photoexcitation of the electrons. The UEDS experiments have revealed that highly anisotropic transient phonon populations are established upon photoexcitation. By accounting explicitly for electron–phonon and phonon–phonon scattering within an ab initio theoretical description of the coupled electron–phonon dynamics, we have demonstrated that this behavior can be attributed to the preferential emission of high energy optical phonons along the zigzag direction of the BP lattice throughout carrier relaxation. This picture is corroborated by the good agreement between the calculated all-phonon structure factors and the measured UEDS intensity throughout the different stages of the lattice dynamics. Our approach can be extended to 2D materials, and could be exploited more broadly in many areas of material sciences and condensed matter physics, ranging from transport to superconductivity phenomena. For instance, it could be employed to reveal energy transfer pathways accross interfaces in van der Waals heterostructures, or to identify specific phonons involved in complex electron–phonon dynamical processes such as polaron formation or phase transitions.

## Methods

### Sample Preparation and Thickness Determination

The thin black phosphorus (BP) flake was obtained by standard mechanical exfoliation performed in air. The samples were then quickly imaged in the optical microscope and subsequently transferred to a load-lock chamber connected to our main experimental chamber in ultrahigh vacuum. We estimate the total exposure to air to be less than 1 h. We found that this method yielded diffraction patterns consistent with previous experimental works.^35^ Given the multilayer nature of the samples (40 nm corresponds to roughly 80 layers), the observed scattering signals predominantly arise from the bulk, as opposed to the oxidized surface layers of the flake. The signature of oxidized or reconstructed layers in the diffraction pattern is dependent on their exact structural arrangement. Such oxidized layers could give rise to a diffuse background in the low scattering vector region, reflecting the absence of long-range order at the surface. In the experimental diffraction pattern, we observe the presence of forbidden reflections (*h* + l = 2*n* + 1). Such forbidden reflections were also observed in previous works, but their origin could not be attributed with certainty.^35^ We postulate that they may be caused by stacking faults or structural deviations at the surface. These additional reflections do not alter the overall agreement between experiment and theory.

The flake thickness was estimated by transmission measurements in an optical microscope in combination with transfer matrix calculations and the optical constants of BP.^17^

### Computational Details

First-principles calculations employed the primitive cell of bulk BP (point group D_2*h*_ and space group *Cmce*) that contains four atoms.^34^ All calculations were performed using the PBE generalized gradient approximation^50^ to density functional theory (DFT). We employed planewaves basis sets and Troullier–Martins norm-conserving pseudopotentials,^51^ as implemented in the Quantum ESPRESSO suite.^52^ The planewaves kinetic energy cutoff was set to 90 Ry and the sampling of the Brillouin zone was performed using a uniform 12 × 10 × 10 **k**-point grid. We determine the interatomic force constants by means of density-functional perturbation theory calculations,^53^ using a 5 × 5 × 5 Brillouin-zone **q**-grid. The full set of phonon eigenmodes and eigenfrequencies was obtained by using standard Fourier interpolation of dynamical matrices on a 50 × 50 × 50 **q**-point grid. Such a dense grid guarantees a fine resolution of the calculated structure factor maps. The phonon band structure over a chosen high-symmetry path is shown in Figure S2 in the Supporting Information.

## Supporting Information Available

Estimation of excited carrier density; diffuse scattering maps: raw data and influence of pump photon energy; computations of the structure factor; dependence on the initial lattice temperature (PDF)

## Notes

The experimental data presented here are available on a data repository.^54^
