# Supplementary material for: Accessing the Anisotropic Nonthermal Phonon Populations in Black Phosphorus
Source: Nano Lett. 2021 Jul 19;21(14):6171–8. doi: 10.1021/acs.nanolett.1c01786 (PMC8323122; doi:10.1021/acs.nanolett.1c01786)
Supplement: Supplementary file 1 — nl1c01786_si_001.pdf [file nl1c01786_si_001.pdf]

# Supporting Information: Accessing the anisotropic nonthermal phonon populations in black phosphorus

Hélène Seiler,*,† Daniela Zahn,† Marios Zacharias,†,‡ Patrick-Nigel Hildebrandt,† Thomas Vasileiadis,†,¶ Yoav William Windsor,† Yingpeng Qi,†,§ Christian Carbogno,† Claudia Draxl,‖ Ralph Ernstorfer,† and Fabio Caruso*,⊥

†*Fritz Haber Institute of the Max Planck Society, Faradayweg 4-6, 14195 Berlin, Germany*

‡*Department of Mechanical and Materials Science Engineering, Cyprus University of Technology, P.O. Box 50329, 3603 Limassol, Cyprus*

¶*Current address: Adam Mickiewicz University, Faculty of Physics, PL 61-614 Poznan, Poland*

§*Current address: Shanghai Jiao Tong University, 800 Dongchuan Road, 200240 Shanghai, China*

‖*Institut für Physik and IRIS Adlershof, Humboldt-Universität zu Berlin, Berlin, Germany*

⊥*Institut für Theoretische Physik und Astrophysik, Christian-Albrechts-Universität zu Kiel, D-24098 Kiel, Germany*

E-mail: seiler@fhi-berlin.mpg.de; caruso@physik.uni-kiel.de

# Estimation of excited carrier density

The incident fluence on the BP flake is $I_{\text{inc}} = 98$ J/m$^2$. The pump was polarized along the *armchair* direction, determined by rotating a waveplate in the pump arm and maximizing the Debye-Waller effect at 50 ps (maximum absorption). Given the highly anisotropic optical absorption of BP, specifying the pump polarization is essential to employ the proper value of refractive index, which matters for the estimation of excited carried density. The complex refractive index of BP along the *armchair* direction at 800 nm is 3.19 + 0.29i, as estimated from a previous work.[1] We use transfer matrices to calculate the transmitted part, T, as well as the reflected part, R, of the incident fluence. This yields the absorbed fluence $I_{\text{abs}} = (1 - R - T) \cdot I_{\text{inc}}$. The carrier density per square centimeter is then:

$$n = 10^{-6} \cdot \frac{1}{E_{\text{ph}}} \cdot \frac{I_{\text{abs}}}{d} \cdot c \cdot \frac{1}{2} = (7.3 \pm 0.9) \cdot 10^{13} \quad \text{electrons/cm}^2,$$

where $E_{\text{ph}}$ is the energy of one pump photon, $d$ is the flake thickness, which we estimate to 39 ± 5 nm, $c = 10.46 \cdot 10^{-10}$ m is the unit cell length in the out-of-plane direction and the factor 1/2 accounts for the two layers per unit cell.

# Diffuse scattering maps: raw data and influence of pump photon energy

In supplementary Figure 1 we show a series of diffuse scattering maps at chosen delays and for different pump photon energies and/or data processing steps. Panels (a-c) and (j-l) correspond to the experimental and theoretical results shown in Figure 3 of the main text, respectively. They are reproduced here for convenience. Panels (d-f) display the diffuse scattering signals obtained when pumping the sample with 0.59 eV photons. The same initial excited electron density is assumed for both the 0.59 eV and 1.61 eV experiments, based on the Bragg peaks' reduction at 300 ps. While the diffuse scattering signatures at 2 ps seem slightly more intense for the 1.61 eV pump, we observe the same qualitative thermalization process in both cases. Since the picosecond dynamics does not seem to depend significantly on the initial carrier distribution after excitation, the assumption of a thermalized electron system for our purposes seems justified. In the main text and in panels (a-f), we show two-fold symmetrized data. This processed data is obtained using the open-source Python environment developed by De Cotret et al.[2] Prior to symmetrization, it was verified that the time-dependence of the elastic scattering for Friedel pairs showed the same dynamics within error margin. The raw data for the 1.61 eV pump are shown in panels (g-i). Comparing panels (a-c) and (g-i), we observe peak splitting effects at large scattering vectors for the symmetrized data. This artifact arises from field distortions of our magnetic lens, which contains aberrations at higher scattering vectors.

# Computations of the structure factor

We perform computations of the *all-phonon* structure factor $I_{\rm all}(\mathbf{Q},T)$.[3,4] We find that taking into account multi-phonon effects is essential for an accurate reproduction of the experimentally observed diffraction patterns of BP seen in Figures 3(a-c) of the main text. The expression for $I_{\rm all}(\mathbf{Q},T)$ reads:

$$I_{\rm all}(\mathbf{Q},T) = N_p \sum_{\kappa\kappa'} f_\kappa(\mathbf{Q}) f^*_{\kappa'}(\mathbf{Q}) e^{-W_{\kappa\kappa'}(\mathbf{Q},T)} \sum_p e^{i\mathbf{Q}\cdot[\mathbf{R}_p+\boldsymbol{\tau}_\kappa-\boldsymbol{\tau}_{\kappa'}]}\, e^{P_{p,\kappa\kappa'}(\mathbf{Q},T)}. \quad (1)$$

Here $N_p$ is the number of $\mathbf{q}$-points used to sample the first Brillouin Zone, $f_\kappa(\mathbf{Q})$ denotes the scattering amplitude of atom $\kappa$, $W_{\kappa\kappa'}(\mathbf{Q},T)$ is the Debye-Waller factor, $\boldsymbol{\tau}_\kappa$ represents the atomic positions and $\mathbf{R}_p$ defines the position vector of unit cell $p$ contained in a Born-von Kármán supercell. The phononic factor, $e^{P_{p,\kappa\kappa'}(\mathbf{Q},T)}$, includes all orders of phonon processes and its exponent is given by:

$$P_{p,\kappa\kappa'}(\mathbf{Q},T) \;=\; \frac{M_0 N_p^{-1}}{\sqrt{M_\kappa M_{\kappa'}}} \sum_{\mathbf{q}\nu} \langle u^2_{\mathbf{q}\nu}\rangle_T \,\mathrm{Re}\Big[\mathbf{Q}\cdot\mathbf{e}_{\kappa,\nu}(\mathbf{q})\mathbf{Q}\cdot\mathbf{e}^*_{\kappa',\nu}(\mathbf{q}) e^{i\mathbf{q}\cdot\mathbf{R}_p}\Big], \quad (2)$$

where $M_\kappa$ and $M_0$ are the atomic and reference masses, and the phonons are described by the eigenmodes $\mathbf{e}_{\kappa,\nu}(\mathbf{q})$ and frequencies $\omega_{\mathbf{q}\nu}$. A key quantity entering the equation of the structure factor is the mean-squared displacement of the atoms due to mode $\mathbf{q}\nu$, defined as $\langle u^2_{\mathbf{q}\nu}\rangle_T = \hbar/(2M_0\omega_{\mathbf{q}\nu})[2n_{\mathbf{q}\nu}(T)+1]$. The time-dependence of the all-phonon structure factor is encoded in $\langle u^2_{\mathbf{q}\nu}\rangle_T$, which is directly related to phonon populations $n_{\mathbf{q}\nu}(T)$. To account for the influence of the nonequilibrium lattice dynamics on the UEDS intensity, we evaluated Eq. (1) at each time snapshot by populating phonons according to the vibrational temperatures obtained from the solution of the time-dependent Boltzmann equation (Figure 4).

# Dependence on the initial lattice temperature

Numerical simulations included in the main text assumed an initial temperature $T^0_{\rm ph} = 100$ K for the lattice. This value coincides with the temperature at which experiments have been conducted. To explore the dependence of the nonequilibrium dynamics on this parameter, we report in supplementary Figure 3 the average vibrational temperature $\tilde{T}_{\mathbf{q}}$ (see main text) for several initial conditions. The first, second, and third rows in supplementary Figure 3 correspond to initial temperatures of 100, 300, and 500 K, respectively, for time delays up to 40 ps following photoexcitation. The initial electronic temperature has been kept fixed at $T^0_{\rm el} = 7000$ K. We note that, irrespective of the initial conditions, the lattice dynamics exhibits an anisotropic enhancement of the vibrational temperature along the Γ-A direction. The influence of the initial temperature is twofold: First, we observe that for larger initial temperatures, the lattice dynamics is characterized by a smaller increase of the vibrational temperature. This behaviour can be attributed to the non-linear dependence on temperature of the Bose-Einstein distribution function, which in turns defines the energy $\Delta E_{\rm ph}$ gained by the lattice throughout the dynamics:

$$\Delta E_{\rm ph} = \sum_{\nu\mathbf{q}} \hbar\omega_{\mathbf{q}\nu}[n_{\mathbf{q}\nu}(T^{\rm fin}_{\rm ph}) - n_{\mathbf{q}\nu}(T^0_{\rm ph})]. \tag{3}$$

Second, we note that for larger initial temperatures less time is required for the lattice to return to thermal equilibrium. The faster thermalization of vibrational degrees of freedom are due to the shorter phonon-phonon scattering lifetimes. This aspect is further illustrated in supplementary Figure 4, where the time dependence of the vibrational temperature $\tilde{T}_{\mathbf{q}}$ is reported for several initial conditions. These findings indicate that lower temperature are more suitable for the investigation of long-lived nonthermal states of the lattice.

# Supplementary Figures

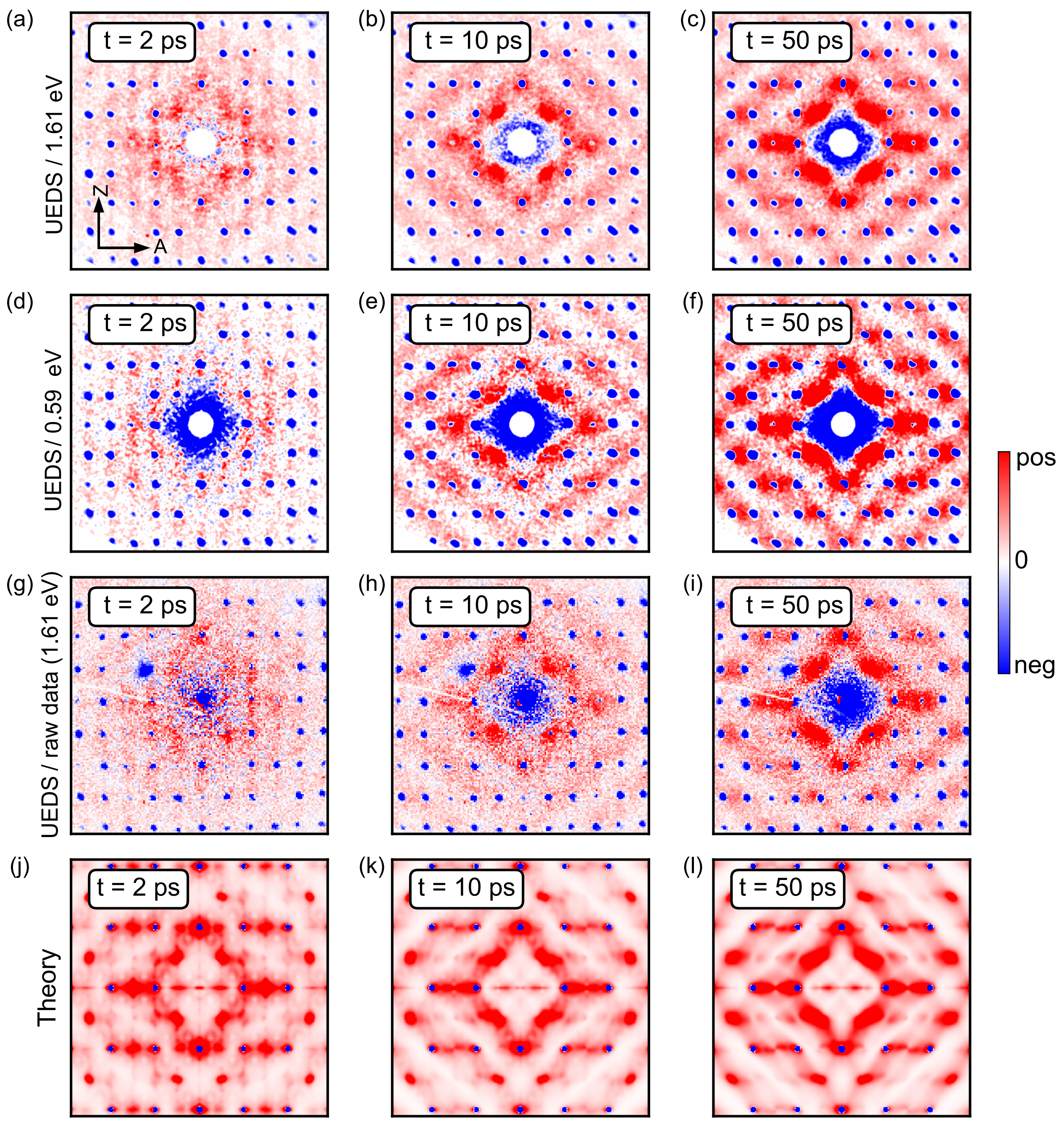


Supplementary Figure 1: Diffuse scattering maps at 2 ps, 10 ps, and 50 ps for different pump photon energies and data processing steps. (a-c) Two-fold symmetrized, 1.61 eV pump. (d-f) Two-fold symmetrized, 0.59 eV pump. (g-i) Raw data (binned), 1.67 eV pump. (j-l) Theory results obtained assuming an excited carried density of $(7.3 \pm 0.9) \cdot 10^{13}$ electrons/cm$^2$.

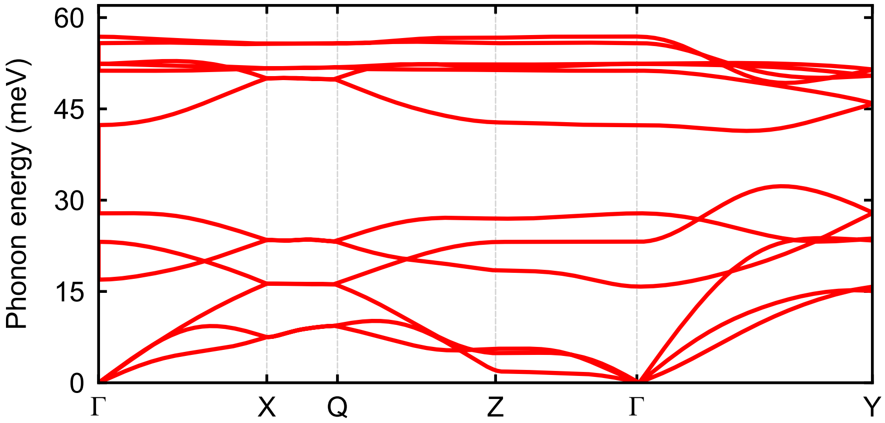


Supplementary Figure 2: Phonon dispersion of BP calculated from density-functional perturbation theory over the Γ-X-Q-Z-Γ-Y path.

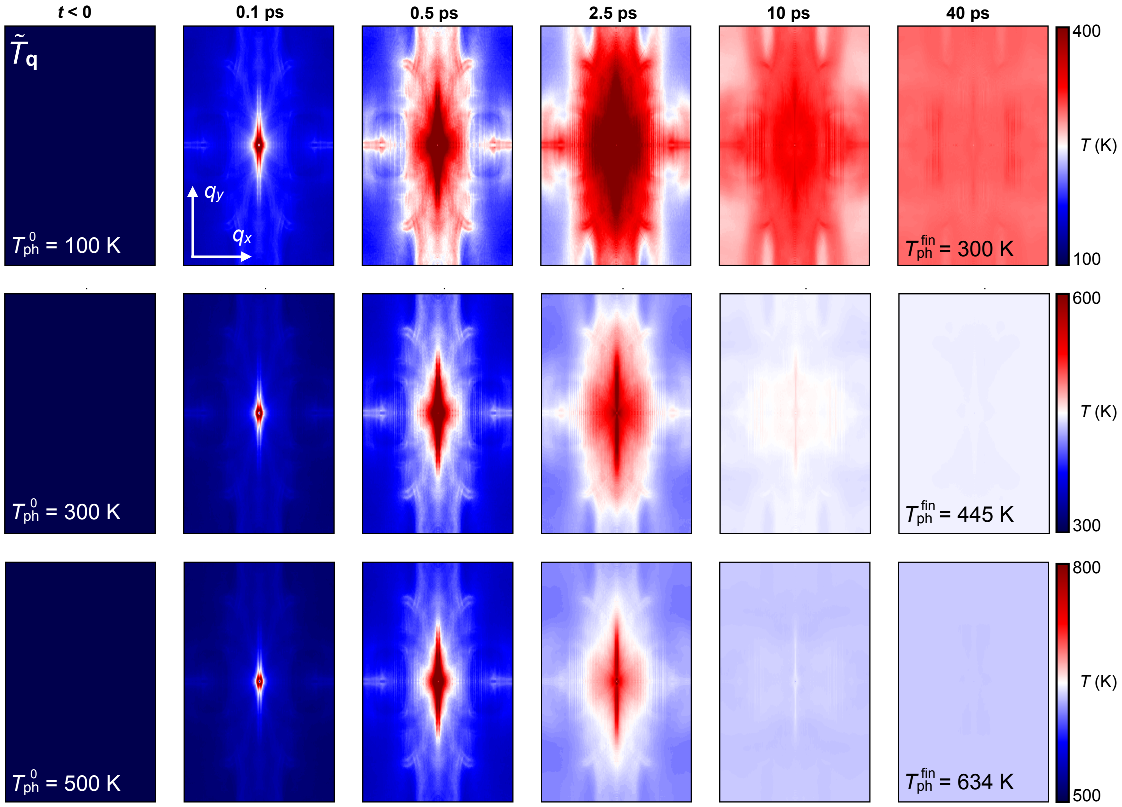


Supplementary Figure 3: Dependence of the simulated average vibrational temperature $\tilde{T}_{\mathbf{q}}$ (see main text) on the initial conditions, for several time delays following excitation. The initial electronic temperature has been kept fixed at $T_{\mathrm{el}}^{0} = 7000$ K. Initial lattice temperatures of 100, 300, and 500 K have been considered in the first, second, and third row, respectively.

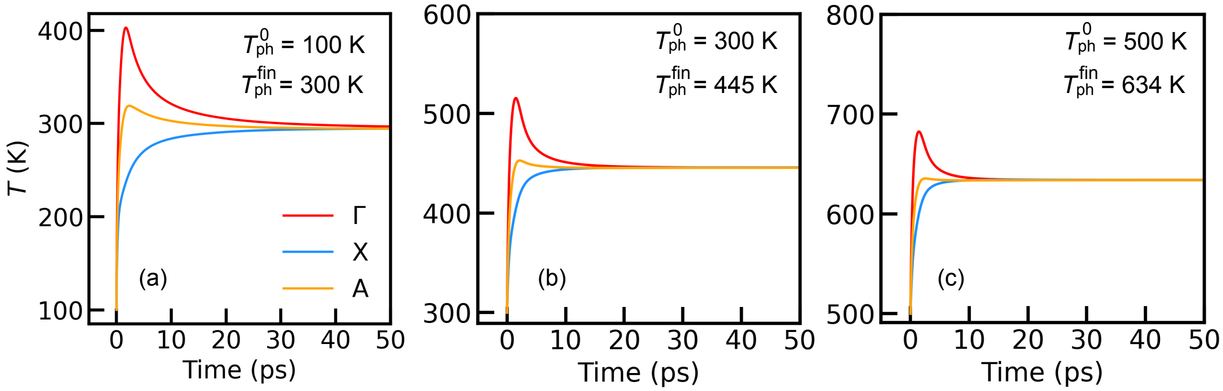


Supplementary Figure 4: Time-dependence of the vibrational temperature $\tilde{T}_{\mathbf{q}}$ at the X, Γ, and A high-symmetry points for initial lattice temperatures of (a) 100 K, (b) 300 K, and (c) 500 K.

# References

(1) Jiang, H.; Shi, H.; Sun, X.; Gao, B. Optical Anisotropy of Few-Layer Black Phosphorus Visualized by Scanning Polarization Modulation Microscopy. *ACS Photonics* **2018**, *5*, 2509–2515.

(2) de Cotret, L. P. R.; Otto, M. R.; Stern, M. J.; Siwick, B. J. An open-source software ecosystem for the interactive exploration of ultrafast electron scattering data. *Advanced Structural and Chemical Imaging* **2018**, *4*, 1–11.

(3) Zacharias, M.; Seiler, H.; Caruso, F.; Zahn, D.; Giustino, F.; Kelires, P. C.; Ernstorfer, R. First-principles calculation of the all-phonon inelastic scattering in solids. **2021**, arXiv:2103.10108. arXiv.org. https://arxiv.org/abs/2103.10108 (accessed July 7, 2021).

(4) Zacharias, M.; Seiler, H.; Caruso, F.; Zahn, D.; Giustino, F.; Kelires, P. C.; Ernstorfer, R. Multi-phonon diffuse scattering in solids from first-principles: Application to 2D $MoS_2$, bulk $MoS_2$, and black Phosphorous. **2021**, arXiv:2104.07900. arXiv.org. https://arxiv.org/abs/2104.07900 (accessed July 7, 2021).
